# Born to Run? Diverse Modes of Epithelial Migration

**DOI:** 10.3389/fcell.2021.704939

**Published:** 2021-08-12

**Authors:** Pengfei Lu, Yunzhe Lu

**Affiliations:** School of Life Science and Technology, ShanghaiTech University, Shanghai, China

**Keywords:** cell polarity, epithelial polarity, apicobasal polarity, collective migration, EMT, extracellular matrix, mechanosensing, mesenchymal-epithelial transition

## Abstract

Bundled with various kinds of adhesion molecules and anchored to the basement membrane, the epithelium has historically been considered as an immotile tissue and, to migrate, it first needs to undergo epithelial-mesenchymal transition (EMT). Since its initial description more than half a century ago, the EMT process has fascinated generations of developmental biologists and, more recently, cancer biologists as it is believed to be essential for not only embryonic development, organ formation, but cancer metastasis. However, recent progress shows that epithelium is much more motile than previously realized. Here, we examine the emerging themes in epithelial collective migration and how this has impacted our understanding of EMT.

## Introduction

The emergence of the epithelium is one of the earliest and most important events during metazoan evolution. By separating an organism from the outside world, the epithelium protected cells within the organism from environmental insults and facilitated the rise and prosperity of multicellularity. Far from being a simple, protective shield or barrier, the epithelium also allowed an organism to actively exchange substances, including nutrients, wastes, gases, etc. with the environment, a basis on which organ systems, including the digestive, respiratory, urinary, and glandular systems emerged during evolution. These organ systems enabled the metazoans to explore previously untapped niches and, eventually, to dominate almost every corner of the planet earth (Dickinson et al., 2011). Today, most major organs of extant animals are epithelial and most human cancers are of epithelial origin, thus highlighting the importance of epithelial function in physiology and pathology.

Historically, the epithelium is considered being immotile. This is in part because of the observation that the epithelium is rich in myriads of cell-cell adhesion proteins, particularly tight junctions (TJs) and adherens junctions (AJs), and focal adhesions which anchor the epithelium to the matrix, i.e., the basement membrane. To migrate, it is believed that the epithelium must first transition to a mesenchymal state via a process called epithelial-to-mesenchymal transition (EMT) (Hay, 1968, 1995). Epithelial-to-mesenchymal transition was subsequently confirmed in several fundamental developmental processes, including the generation of mesoderm and neural crest (Newgreen et al., 1979; Thiery et al., 1982). Research in the 1990s further implicated EMT with pathologies like fibrosis and cancer (Valles et al., 1990; Miettinen et al., 1994). The notion that EMT may be responsible for these pathologies such as cancer metastasis further catapulted EMT to one of the most intensely researched areas in not only developmental biology but in molecular biology and cancer biology as well in the past decade (Yang et al., 2020).

Tremendous progress has been made since the initial description of EMT. We now know, for example, the epithelium is much more motile than expected, especially during embryonic development and organ formation where large scale migration such epiboly and convergent extension during gastrulation have all been well documented. Due to space constraints, these types of migration will not be discussed in detail in the current review. Instead, we will focus on directional movements of epithelial cells, where recent studies show that occur collectively rather than individually as it was previously believed (Cheung et al., 2013, 2016; Wrenn et al., 2020). We first discuss recent progress in epithelial collective migration and then how it has impacted our current understanding of EMT.

## The Evolving Concept of Epithelial-To-Mesenchymal Transition

At the core of the current debates on EMT lie the very definitions of what makes up an epithelial or a mesenchymal state, and what the most important changes are, at both the cellular and molecular levels, that occur during the EMT process.

### What Makes an Epithelium?

**FIGURE 1:**
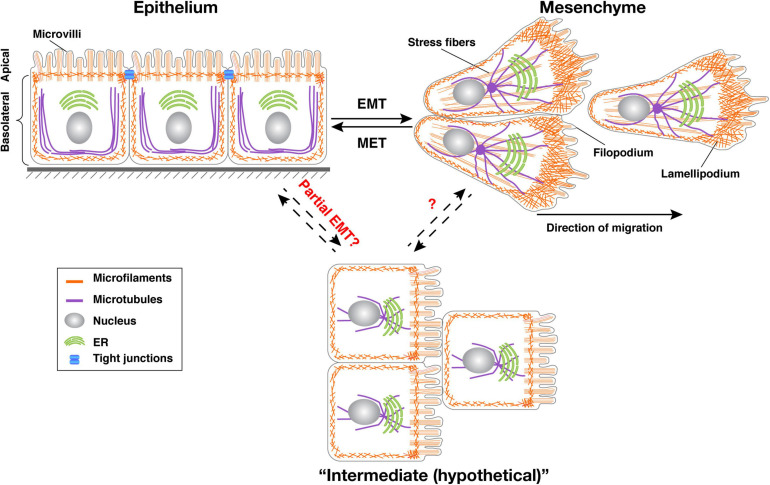
Transitions between epithelial, mesenchymal, and intermediate cell states. Typical vertebrate epithelium structure with apicobasal polarity. Tight junctions separate the apical domain from the basolateral domain. Upon EMT, cells lose apicobasal polarity, reorganize their cytoskeleton, and become motile. Increasing evidence shows that cells undergo collective migration rather than individual migration after EMT. Both epithelial and mesenchymal states are plastic and mesenchymal cells can return to the epithelial state in both development and cancer situations through a process called MET. It is believed that cells, especially in cancer situations, can enter a “partial EMT” state where they express mixed epithelial and mesenchymal gene signatures. Microfilaments are indicated in red, microtubules in green. The position of the nucleus (light blue oval) is also shown.

Epithelium can be defined at several levels. At the functional level, as mentioned above, an epithelium provides tissue with a protective barrier and substance-exchange roles. Depending on their cell shapes, the mature epithelium can be categorized into simple epithelium, including squamous, columnar, or cuboidal epithelium; stratified epithelium, which contains multiple cell layers; and compound epithelium, which has a mixture of one or more of the above features. We will not differentiate epithelial migration based on these categories of the mature epithelium as the current literature is primarily focused on the developing epithelium. Regardless of their categories, one of the most salient feature of epithelium is its polarity, i.e., its plasma membrane is organized into two discrete regions: one facing the environment, called the apical domain and another facing the inside of the tissues, called the basolateral domain (Figure 1) (Bryant and Mostov, 2008; Mellman and Nelson, 2008). With these two distinct domains, membrane proteins of various functions, for example, ion or metabolite transporters can be differentially localized to either the apical or the basolateral domain, and selectively regulate the exchange of substances between the environment and the organism (St Johnston and Ahringer, 2010).

At the structural level, apicobasal polarity is made possible in part by the unique presence and organization of various membrane junctional components. Specifically, epithelial polarity is manifested by the establishment of the apical junctional complex (AJC), which includes the TJs in vertebrates, or septate junctions in invertebrates, and AJs. Tight junctions, which are composed of several families of transmembrane proteins, including Claudins and junctional adhesion molecules (JAMs), are organized into a tight seal that prevents the free diffusion of proteins and lipids between apical and lateral surfaces. They also make up an important selective barrier regulating the diffusion of molecules through the paracellular space. Interestingly, although Occludin was the first integral TJ resident component identified, genetic studies have shown that it is not essential for TJ function but it plays other important cellular functions (Bryant and Mostov, 2008).

Basal to tight junctions in vertebrates, but apical to septate junctions in invertebrates, are AJs, which form an adhesive belt encircling each epithelial cell just underneath the apical surface. AJ transmembrane components include cadherins, most notably E-Cadherin, which provide cohesion between cells of the epithelial sheet. Although all epithelia possess apicobasal polarity, they may differ in the kind, amount, affinity of their adhesion molecules for other cells and matrix. They may also differ in the absence or presence, and complexity of the ECM (Campbell and Casanova, 2016).

### EMT in Normal Development and Pathologies

Epithelial-to-mesenchymal transition (EMT) was first described by Elizabeth Hay in the 1970s while studying chick embryogenesis using *in vitro* explant (Hay, 1968, 1995). It was subsequently confirmed in the context of neural crest formation (Newgreen et al., 1979; Thiery et al., 1982), heart valve formation (Markwald et al., 1977), and Mullerian duct regression during kidney development (Trelstad et al., 1982). Additional classic examples of EMT also include mesoderm formation during gastrulation (Leptin, 1991), sclerotome formation from the somite (Christ and Ordahl, 1995), and wound healing (Yang et al., 2020). It is generally considered including several distinct steps: first, EMT signals, often extrinsic paracrine factors and/or intrinsic transcription factor activation, stimulate target epithelial cells; second, epithelial cells lose apicobasal polarity and downregulate E-Cadherin expression; third, epithelial cells transition to mesenchymal cells and start to migrate.

The reverse process of EMT, known as a mesenchymal-epithelial transition (MET), also occurs during development, for example during secondary neurulation and nephron formation of kidney development (Stark et al., 1994). Over the past twenty years, the EMT research field has grown exponentially, thanks in part to the realization that EMT may be activated during pathological conditions such as cancer development and tissue fibrosis (Valles et al., 1990; Miettinen et al., 1994). Indeed, it is believed that loss of epithelial polarity is a cancer hallmark and EMT is an essential step during cancer metastasis (Humbert et al., 2003; Bilder, 2004; Lee and Vasioukhin, 2008; Hanahan and Weinberg, 2011).

Much of this research effort has focused on the molecular nature of the “EMT program”. Various paracrine factors, including TGF-beta, and transcription factors, such as Snail, Slug, Zeb1 and Zeb2, have been identified (Leptin, 1991; Yang et al., 2004; Stemmler et al., 2019). Downstream changes of these paracrine and transcription factors during EMT have also been described (Yang et al., 2004). Together, these changes have been considered as a part of the EMT program and intense research effort has been dedicated to their description under different developmental and pathological conditions.

### Limitations of the EMT Concept: Partial EMT Comes to the Rescue

Much progress has been made since the initial description of EMT. Although cells are thought to migrate individually as single cells following EMT, epithelial cells are increasingly found to migrate together using collective migration (Cheung, 2016). Indeed, recent studies show that cancer cells undergoing collective migration can metastasize more efficiently and survive better at distant sites (Cheung et al., 2013, 2016; Wrenn et al., 2020). However, the limitations of the EMT concept have also started to emerge. For example, although the downregulation of cell-cell adhesion was considered to be a prerequisite for the EMT process, increasing evidence suggests that this might not be the case. Specifically, E-cad is instead upregulated during Drosophila border cell migration (Cai and Montell, 2014; Cai et al., 2014). In breast cancer, E-cad downregulation did not promote metastasis but instead triggered cell deaths. In this case, E-cad functions similarly to an oncogene and promotes cancer development (Padmanaban et al., 2019).

Furthermore, using molecular signatures of EMT programs, a growing number of studies show that cell conversions to either the epithelial or mesenchymal state are often not “complete” (Tarin et al., 2005; Thompson et al., 2005; Fischer et al., 2015; Zheng et al., 2015). To reconcile these discrepancies, the phrase “partial EMT” has been coined to describe the cell states that are intermediate epithelial/mesenchymal states (Nieto et al., 2016). But the exact definition of what constitutes complete or partial EMT is controversial and is intensely debated at present (Aiello et al., 2017; Ye et al., 2017; Yang et al., 2020). Due to space constraints, here we focus on one of the most fundamental traits that differentiate epithelial and mesenchymal states, i.e., their ability/inability to migrate.

## Mechanism of Directional Migration of Single Cells

Directional cell migration is a complex and highly regulated process requiring constant crosstalk between cells and their environment (Petrie et al., 2009; Krause and Gautreau, 2014). Cells must be able to perceive various environmental cues, often in the form of a gradient, and orient themselves in such a way that they can move directionally toward the signal (Petrie et al., 2009). Here, we first discuss migration of single cells, not only because it is one of the ways that epithelial cells migrate upon completing EMT and that it is the most well-understood migration process, but also because epithelial collective migration faces similar issues as single cells and, at the individual cell level, share many similarities during migration (Table 1).

**TABLE 1:** Comparison of direction determination and force generation between single cell migration and collective cell migration.

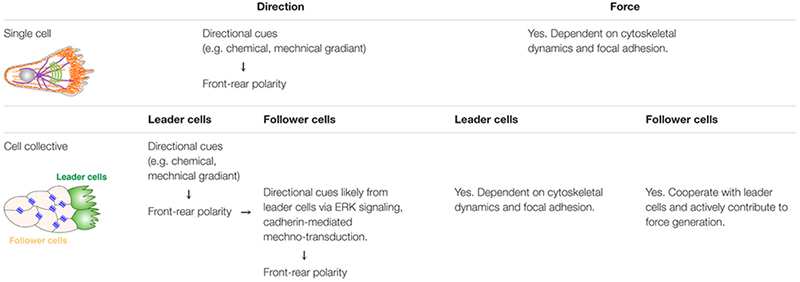

### Determination of Front-Rear Polarity During Single-Cell Migration

Regardless of whether cells migrate individually as single cells or collectively as a cohort, they are confronted with two issues that must be resolved: first, how is their direction of migration determined? Second, where does the force come from to drive migration? To solve the first issue, the direction of migration is determined by a front-rear polarization process (Haeger et al., 2015). Specifically, upon stimulation by an external cue, be it chemokines, growth factors, mechanical forces, or electrical fields, a cell reorients its plasma membrane domains, cytoskeletal organization, nucleus, and organelles, in such a way that is most suited for directional migration. When the cell involved is of epithelial origin, it means that it must first convert apicobasal polarity into front-rear polarity (Etienne-Manneville, 2004; St Johnston and Ahringer, 2010).

**FIGURE 2:**
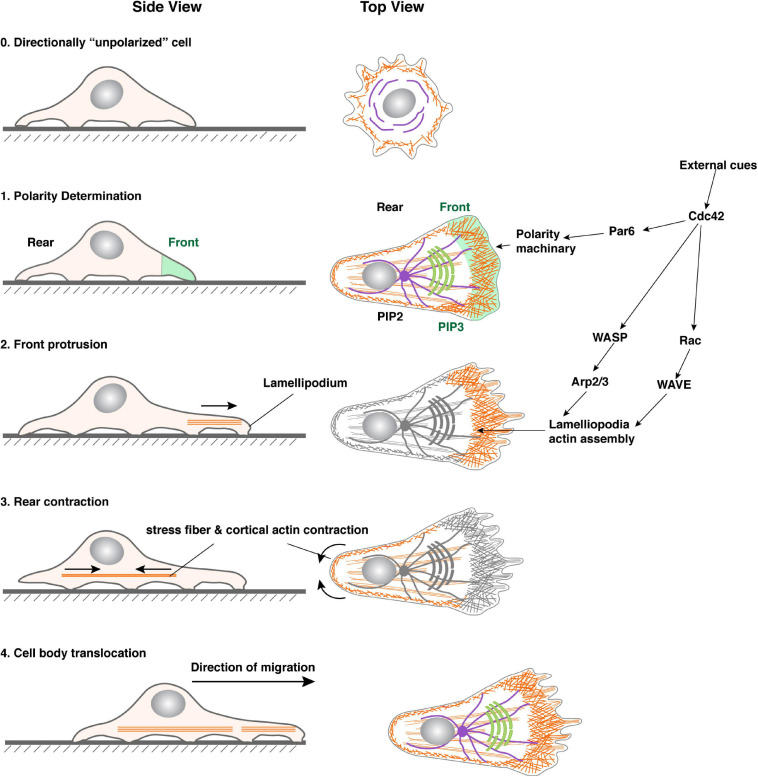
Steps in single-cell migration. Diagram with both side and top views of a cell before and during migration to illustrate signaling events leading to polarity determination, microfilament dynamics, and morphological changes of the cell. For illustration purposes, a cell is theoretically “unpolarized” before being stimulated by an external cue (0). Upon stimulation, the area with the highest Cdc42, Par6, and downstream signaling activities becomes the front, which is marked by an increase in PIP3 levels in the membrane and focal adhesions assembly (1). As a result, lamellipodia formation starts in the front, leading to membrane forward protrusion, where new focal adhesions can be made (2). Concordantly, the rear of the cell undergoes a contraction process (arrowheads) involving actomyosin activities along the cell periphery and in the stress fibers (3). The cell retracts when focal adhesions in the rear break apart due to the contraction force and, as a result, the cell body translocates forward. The net results of the above steps are a forward movement of the cell.

Although the molecular details have yet to be completely worked out and there appear to be some variations depending on the models examined, the general principle seems to be quite conserved (Figure 2). Thus, upon stimulation by an external cue, a cell is locally activated by its ECM adhesion receptors, receptor tyrosine kinases (RTKs), or G-protein- coupled receptors (Affolter and Weijer, 2005; Petrie et al., 2009; Krause and Gautreau, 2014). This local activation of these receptors then activates GTPases of the Rho family, particularly Cdc42 and Rac1, and phosphoinositide 3-kinase (PI3K) (Ridley et al., 2003; Srinivasan et al., 2003; Hammond and Hong, 2018). Activated Cdc42 then turns on the Par family of the polarity machinery to initiate the polarization process (Etienne-Manneville, 2004).

As discussed below, Par6, Par3, and atypical PKC (aPKC) family proteins were initially discovered in cell polarity during *Caenorhabditis elegans* embryonic development and, together with Cdc42 GTPases, regulate cell polarity in different cell behavior (St Johnston and Ahringer, 2010). They often work hand-in-hand, and often antagonistically, with two other groups of proteins in the polarity machinery, namely the Crumbs complex and the Scribbled/Discs Large/Lethal giant larvae complex. Indeed, many of the components of these two other complexes have been shown to play a role in single-cell migration (Ridley et al., 2003).

As a result of the establishment of this signaling, front-rear polarity is set up: on the cell membrane surface, PIP3 marks the front, in part due to localized activity of PI3 Kinase in the front, and PIP2 marks the rear. At the level of cytoskeleton arrangements, the lamellipodia-based actin network lies in the front, whereas cortical actin and stress-fiber-based actin bundles mark the rear (Ridley et al., 2003; Vaidziulyte et al., 2019). The microtubule network is also polarized, with the microtubule organizing center (MTOC) lining up with the direction of migration either directly in the front or the back of the nucleus (Figure 2) (Zhang and Wang, 2017).

### Force Generation During Single-Cell Migration

Once front-rear polarity has been set up, a cell moves forward primarily by two forces, both of which depend on actin filament but with important differences. Specifically, in addition to setting up a front-rear polarity, activated Cdc42 promotes an Arp2/3-dependent actin assembly process. Because Arp2/3 is involved in actin nucleation and the plus ends of actin filament are anchored at the focal adhesions, the actin polymerization process is forward-oriented and produces a network of filaments protruding into the front, an extension often referred to as the lamellipodia (Figure 2) (Pollard and Borisy, 2003; Suraneni et al., 2012). Because the action network is fixed concerning the substratum, the front membrane is pushed out as the filaments elongate. This process is very similar to the movement of the bacterial pathogen Listeria, which moves within a cell and sometimes through a cell by “riding” on the polymerizing actin tail (Bravo-Cordero et al., 2013).

Contrary to the front where Cdc42 and Rac1 activities are high, another GTPase Rho is high at the rear of the cell, partially owing to negative feedback signaling between Rac/Cdc42 and Rho (Goicoechea et al., 2014). Activated Rho, through its main effector ROCK, promotes actomyosin activity by ROCK-mediated phosphorylation of the myosin light chain. As a result, the concerted contraction in both the stress fibers and the cortical actin leads to retraction of the rear of the cell and translocates the cell body forward (Vicente-Manzanares et al., 2009) (Figure 1). In addition to regulating direction and force generation, there are some other aspects of cell migration that are also involved. They are the focus of many excellent reviews (Petrie et al., 2009; Krause and Gautreau, 2014) and will not be discussed further here.

## Determination of Front-Rear Polarity of the Migrating Collective

Like migrating single cells, a cohort of cells also needs to determine the direction and force of migration. Unlike single cells, however, these two issues not only need to be addressed at the single-cell level but at the level of the cohort, as discussed below.

### Leader Cells Define the Front and Drive the Migrating Collective

**FIGURE 3:**
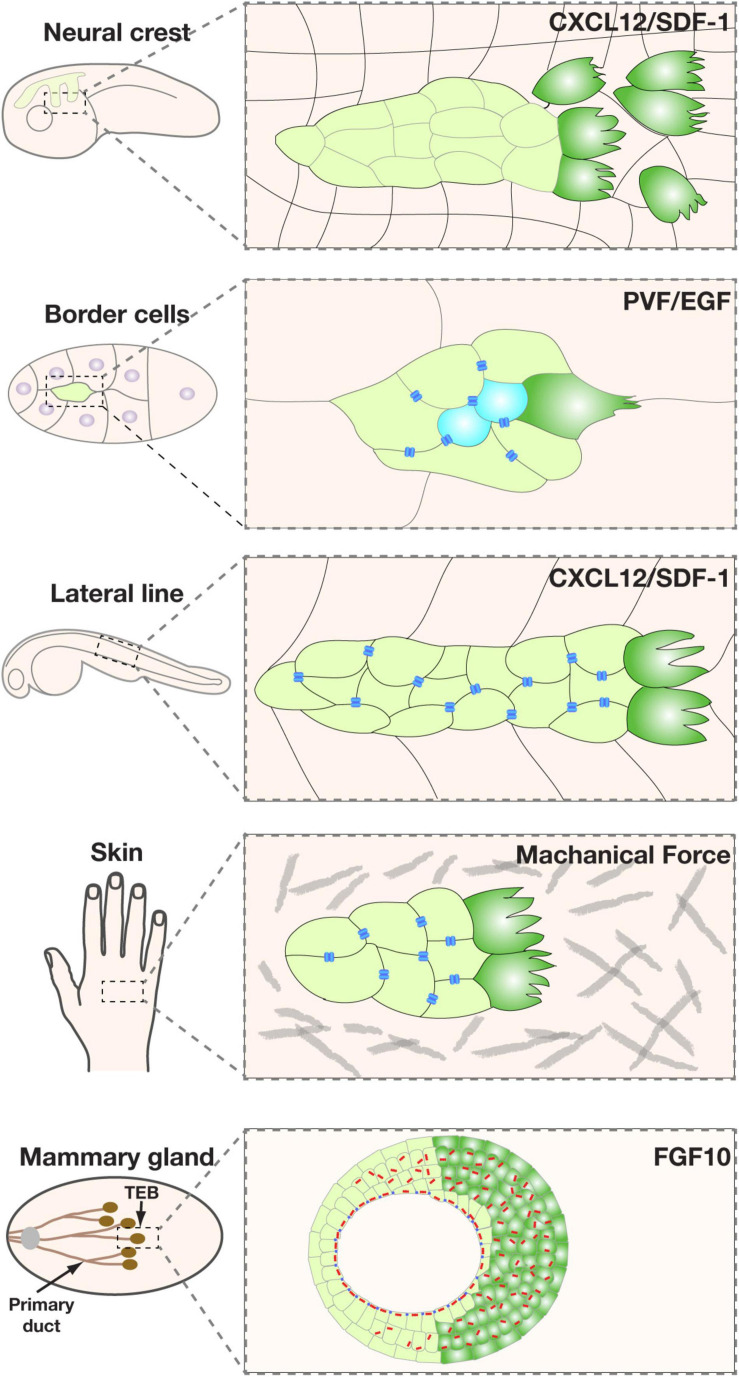
Representative models of collective migration. Front-rear polarity in most of these models, including the *Xenopus* neural crest, *Drosophila* border cells, zebrafish lateral line, and mouse mammary gland, is triggered by a chemoattractant indicated, except for the wound healing model (using human skin as an example), where the external cue is thought to be mechanical forces. Leader cells are colored in green. Blue bars indicate the boundary of the apical and basolateral domains, which in vertebrates is where tight junctions locate, and in invertebrates is the subapical complex. They denote the presence of apicobasal polarity and the epithelial state. Note that neural crest cells do not have the blue bars because they are thought to have undergone a complete EMT and have lost the apicobasal polarity. Leader cells are colored in green.

One of the earliest morphological signs of front-rear polarity during collective migration is the formation of actin-rich filopodia and lamellipodia at the leading edge of the migrating unit (Haeger et al., 2015). Interestingly, although every cell that migrates individually forms these kinds of cellular extensions pointing toward the external cues, only one or a few cells at the migration front do so when they migrate as a collective (Friedl and Gilmour, 2009; Petrie et al., 2009). These cells are often referred to as “leader cells” because of their location at the leading position, whereas those in the rear are referred to as “follower cells” (Khalil and Friedl, 2010; Theveneau and Linker, 2017) (Figure 3).

In all of the collective systems examined, leader cells are not only a manifestation of directionality but also the source of traction forces that power the migration process (Friedl and Gilmour, 2009; Petrie et al., 2009; Haeger et al., 2015). In both the *Drosophila* trachea during embryonic development and air sacs during larva stages, directional migration is driven by leader cells in response to FGF cues and is essential for patterning branch (Lu et al., 2006). Likewise, leader cells also exist at the invasion front of other migrating collectives, including fly border cells, vertebrate neural crest, zebrafish lateral lines, etc., and are responsible for “pulling” follower cells forward during directional migration (Friedl and Gilmour, 2009; Petrie et al., 2009) (Figure 3).

It has long been assumed that vertebrate epithelia, especially those from branched organs, including the lung, kidney, and mammary gland, undergo directional migration as a part of their ontogeny (Ewald et al., 2008; Lu and Werb, 2008; Lu et al., 2008). The assumption is in part based on the observation that invertebrate and vertebrate branching systems, which are non-homologous structures, share a surprising amount of cellular and molecular events, or “deep homology” during organ formation (Lu et al., 2006; Lu and Werb, 2008). Interestingly, our recent work showed that leader cells in the mammary gland are a dynamic population and move faster and more directionally toward the FGF10 signal than follower cells, owing partly to their intraepithelial protrusions toward the signal. We show that a leader cell in the migrating mammary epithelium might “pull” or “push” the collective depending on its relative position in the collective and whether there are cells in front of them. It thus uses a novel mechanism during directional collective migration (Lu et al., 2020) (Figure 3).

### Specification of Leader Cells

Many of the aforementioned external cues, including RTK ligands, are involved in specifying leader cells. In fly trachea and air sacs, for example, mesodermal cells express Branchless (Bnl/Fgf), which causes migration and branch initiation of adjacent epithelial cells expressing the receptor Breathless (Btl/Fgfr) (Ghabrial et al., 2003). Bnl/Fgf and its downstream events determine whether a cell becomes a leader cell, aka tip cell, or a follower cell, aka stalk cell. Although all tracheal epithelial cells express Btl/Fgfr and respond to Bnl/Fgf, they compete for the ligand, and the cell with the highest Bnl/Fgf signaling activities becomes the leader cell (Ghabrial and Krasnow, 2006). Likewise, EGF and PVF are involved in specifying leader cells in the fly border cell system, while CXCL12/SDF-1 is involved in leader cell specification in neural crest and zebrafish lateral line systems (Lu et al., 2006, 2008; Friedl and Gilmour, 2009) (Figure 3). Furthermore, in a 2D-migration model, Notch1-Dll4 signaling has been shown to play a role in determining leader cell fate (Riahi et al., 2015; Wang et al., 2017).

Moreover, the formation, maintenance, and subsequent migration are under local and sometimes global regulation. In the fly trachea, the number of branches is regulated by mutual inhibition, whereby epithelial cells inhibit each other to take the leading position as they compete for branch-inducing factors. In both the fly trachea and the vasculature, such mutual inhibition depends on Notch signaling. Once leader cells have been determined, they are the only cells of the primary branch that depend on Bnl/Fgf signaling; the remaining follower cells follow leader cells in a Bnl/Fgf-independent manner (Ghabrial and Krasnow, 2006). Likewise, in both fly air sacs and the mammary gland, FGF signaling activity is necessary for cells to remain in the leader but not in the follower position (Lu et al., 2008). Interestingly, in addition of having differential gene expression, recent studies show that energetic regulation is distinct in leader and follower cells. Thus, in a 2D breast cancer invasion model, leader cells exhibit higher glucose uptake than follower cells. Moreover, their energy levels, as revealed by the intracellular ATP/ADP ratio, must exceed a threshold to directionally migrate (Zhang et al., 2019).

Recent studies have highlighted the essential roles of mechanosensing in collective migration. Indeed, Merlin, a Hippo signaling component has been shown to be an essential component of a mechano-transduction pathway guiding collectively migrating epithelium (Das et al., 2015). Consistent with this notion, in addition to responding to chemical gradient, a cell collective may respond to other forms of gradient, including mechanical gradient to determines its leader cells (Vishwakarma et al., 2018). Interestingly, the presence of a mechanical gradient alone is sufficient to trigger directional epithelial migration both *in vitro* and *in vivo* (Camley and Rappel, 2017; Barriga et al., 2018).

In the mammary gland, leader cell formation is a multistep process. First, front-rear polarity is set up when front epithelium undergoes increased cell proliferation and thickening. In the second step, the front epithelium becomes stratified and partially loses apical-basal polarity, leading to the generation of leader cells. In the third step, leader cells, which are a dynamic rather than a stable population and they move faster and more directionally than rear follower cells, extend their intra-epithelial protrusions along the direction of the FGF10 gradient, thus generate a coordinated force to power epithelial migration toward FGF10 signal. In the mammary gland, epithelial geometry determines the potential branching sites due to self-inhibition. In this case, though, self-inhibition depends, at least in part, on TGF-b signaling activities, suggesting that mutual inhibition is also at work in the branching epithelium of vertebrates. Interestingly, in mammals, physical interactions between epithelial and mesenchymal cells may also enable migration in some contexts (Labernadie et al., 2017). Whether such interactions provide directional cues to epithelial cells via chemical gradient or mechanics remains unclear at the present.

Once determined, leader cells are generally thought to directionally migrate using a similar set of molecular machinery as single cells. Indeed, many of the polarity genes and cytoskeletal regulators essential for single cell migration are also required by leader cells during collective migration (Haeger et al., 2015; Yamaguchi et al., 2015).

### Front-Rear Polarity Is Regulated by an Evolutionarily Conserved Machinery Essential for Diverse Cell Behavior

Comparing to studies on single-cell migration, research in collective migration is relatively recent. Emerging evidence shows, however, that a similar set of molecules determine front-rear polarity in leader cells those in single-cell migration. For example, they all involve Cdc42 GTPase and interactions with the three polarity complexes containing the Par, the Crumb, and the Scribble complexes (Etienne-Manneville, 2004; Polgar and Fogelgren, 2018).

**FIGURE 4:**
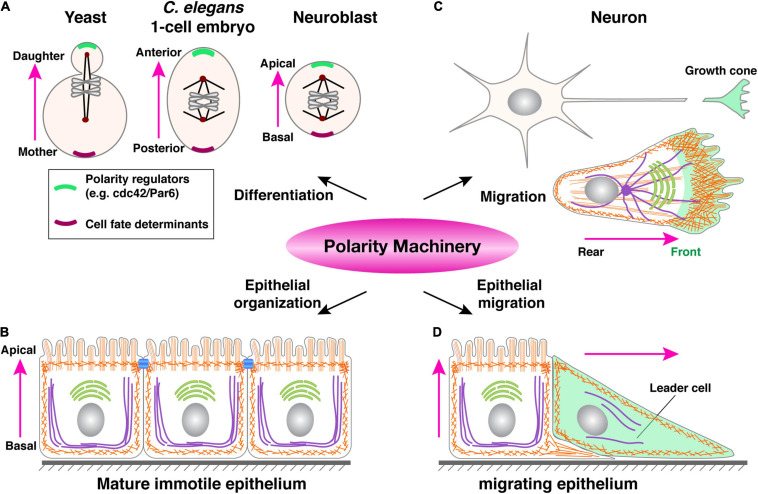
A common polarity machinery regulates distinct cell behavior. **(A)** Yeast cell division, anterior-posterior determination during one-cell stage embryo development of *C. elegans*, and neuroblasts differentiation can all be viewed as different forms of cell fate determination. They all involve the asymmetric distribution of cell fate determinants (purple) upon polarity determination by regulatory machinery (green). **(B)** Mature immotile epithelium whose most salient feature is apicobasal polarity, as indicated by cell morphology and the presence of tight junctions (blue bars). **(C)** A migrating single cell, whose front is colored in green. Note that a neuron can be viewed as a specialized migrating cell, with its growth cone being the cell front, capable of moving and sensing the environment. **(D)** A migrating epithelium with a leader cell (green) and a follower cell, the latter of which still has typical apicobasal polarity. Pink arrows indicate polarity directions.

Some of these polarity regulators, including Cdc42 and the PAR3/PAR6/aPKC complex, are best known for their role in controlling the anterior-posterior axis during the one-cell stage of the *C. elegans* embryo (St Johnston and Ahringer, 2010; Pichaud et al., 2019). As a classic model of “mosaic development”, the fate of each cell at every stage during *C. elegans* embryonic development is fixed, i.e., predetermined by the presence or absence of cell fate determinant(s). Thus, an essential role that these polarity proteins play is to, upon fertilization, set up an intracellular asymmetry, or cell polarity, so that fate determinants, be they proteins or RNAs, could be unevenly distributed to one side of the cell and, upon cell division, one of the two daughter cells (Figure 4A) (Macara and Mili, 2008; Roignot et al., 2013).

Subsequent studies showed that these proteins are widely conserved and dedicated regulators of polarity in animal cells. In keeping with the notion that cell polarity is essential for fate determination in a wide range of metazoan cells, many of the polarity machinery are well known for their role in as distant as the fly and mammalian neuroblasts and unicellular eukaryotes such as yeast cells (Figure 4A) (St Johnston and Ahringer, 2010). Retrospectively, it is remarkable that this same set of polarity regulators also control the social aspect of cell behavior during epithelial formation. In this case, cells coordinate their polarities in such a way that their apical domains all point toward the lumen, while basal domains to the basement membrane, thus constituting apicobasal polarity (Figures 4B–D) (Bilder, 2004; Roignot et al., 2013).

Importantly, polarity proteins are essential for AJC formation as, in the absence of any member of the PAR, CRB, or Scrib complexes, TJ formation is defective (Bilder et al., 2000; Bilder, 2004; Roignot et al., 2013; Overeem et al., 2015; Meiring et al., 2020). However, the precise hierarchy of recruitment and interplay between polarity proteins and AJC components during epithelial polarization remains poorly understood. Furthermore, there appear to be different levels of complexity in terms of how epithelial polarity is regulated. For example, while the loss of Scribble alone is sufficient to disrupt epithelial polarity in fly follicular cells, it is not so in the vertebrate epithelium or the fly intestinal epithelium. It turns out that, in addition to Scribble, several other proteins, including Erbin and Lano also play a similar function in the vertebrate epithelium (Choi et al., 2019; Schmidt and Peifer, 2020), and thus the confirmation of the role of Scribble complex in vertebrate epithelium will have to await the simultaneous removal of these other family members as well.

An important question is why a common, evolutionarily conserved machinery regulates cell behavior as diverse as differentiation, migration, and epithelial organization (Figure 4). While at the first glance, one may argue that molecular conservation of these diverse cell behavior or states indicates an evolutionary co-option, in which the same genes are used for different functions. Upon further examination, however, a more likely possibility is that, despite being seemingly diverse, these different cell behaviors all require a cell to be intrinsically asymmetric so that a cell can adjust its behavior more readily depending on the changing environment (Nance and Zallen, 2011). Such an asymmetry, as manifested by polarized formation and distribution of the cytoskeleton, organelles, plasma membrane, etc. is best achieved by using the same set of protein components, which constitutes common polarity machinery (Polgar and Fogelgren, 2018).

## Followers, but Not Passengers

At present, the prevailing view is that leaders determine migration direction and provide traction force to drive the migration process. However, increasing evidence suggests that, at least in certain models of collective migration, follower cells are not just passive passengers; rather, they cooperate with both leader cells and their neighboring follower cells, and actively participate in the migration process (George et al., 2017).

### Follower Cells Participate in “Supracellularity”

**FIGURE 5:**
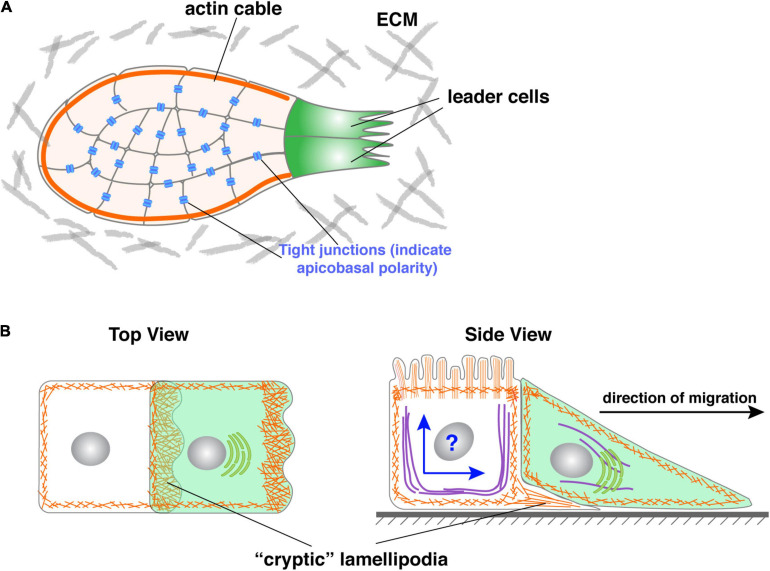
Roles of follower cells during collective migration. **(A)** Periphery follower cells in some models, including neural crest, form actin “cable” whose contraction could propel the forward movement of the collective. Note that actin cable could be a misnomer as it might not represent what the actin-polymer looks like in 3D. **(B)** Follower cells in some models have been shown to form cryptic lamellipodia, which can generate force and promote collective migration. However, as shown in the side view, an essential question is how the follower solves the potential conflict of having two polarities, namely apicobasal and front-rear polarities simultaneously in the same cell when they are mutually exclusive in all other models examined.

One way follower cells may cooperate with leader cells is by working in unison with cells in the entire collective as though they were one giant cell or a “supracell” (Friedl and Mayor, 2017; Venhuizen and Zegers, 2017). This could be accomplished by rearranging their cellular components in such a way that their microfilaments, microtubules, etc. are coherently organized as in a single cell (Figure 5A). For example, it was recently reported that the periphery follower cells of the migrating neural crest form a continuous actomyosin “cable” chain that morphologically resembles the cortical actin in a single cell (Figure 5A) (Shellard et al., 2018). This has also been observed in other *in vitro* and *in vivo* settings in which peripheral follower cells assemble supracellular actomyosin cables at their outward-facing membrane domain (Jacinto et al., 2001; Hidalgo-Carcedo et al., 2011; Lucas et al., 2013; Reffay et al., 2014).

The supracellular actomyosin cable serves at least two important functions during collective migration: first, its contraction can generate force to directly power the forward movement of the collective. During neural crest migration, for example, a disruption of the supracell actin cable abrogates migration, whereas its ectopic activation is sufficient to power collective migration without activation of and input from leader cells. This is not unlike the contraction of cortical actin that powers the retraction of the rear of the cell during single-cell migration that we mentioned earlier (Figure 2) (Shellard et al., 2018). Second, the actin cable also prevents ectopic leader cell formation. In its absence, for example, ectopic leader cells form in fly border cells and, as a result, border cells fail to migrate to their destination (Ramel et al., 2013). Ablating this cable leads to recoil, indicating that it is under tension, and causes the formation of lamellipodia from peripheral follower cells (Ramel et al., 2013; Reffay et al., 2014). Finally, it is important to note that, in addition to unite and engage follower cells, supracellular actomyosin cables also exist in leader cells and play an important role in certain contexts of wound healing and epithelial closure (Vedula et al., 2015). It is thus an essential mechanism to unite all of the cells, both leader cells and follower cells, in the collective to form a supracell.

At present, the mechanism by which a supracell forms the actin cable remains largely unclear. However, recent evidence suggests that it may be similar to what regulates the formation of cortical actin and stress fibers in single cells, both of which depend on Rho-ROCK signaling but are inhibited by cell-cell adhesions, including E-Cad and Discoidin domain receptor 1 (DDR1)-based signaling (Wennerberg et al., 2003; Hidalgo-Carcedo et al., 2011). This potential mechanism explains why supracellular actin cable is formed only at the outside, rather than inside or other cortical domains of the periphery follower cells; it also explains why it is absent from inside follower cells as well (Friedl and Mayor, 2017; Venhuizen and Zegers, 2017).

### Evidence That Follower Cells May Generate Force

As mentioned above, the main school of thought in the field of collective migration currently holds that leader cells both provide the driving force and determine the direction of migration (Trepat et al., 2009; Kim et al., 2013). Emerging evidence suggests that, at least in some model systems, follower cells can also generate force and thus actively drive the forward movement. Indeed, Farooqui and Fenteany showed in the Madin-Darby Canine Kidney cells wound-healing assays that follower cells, rows behind leader cells, also form lamellipodia toward the direction of migration. Unlike those formed by leader cells, these follower cells’ lamellipodia are “hidden” under the cells in front of them and thus have been referred to as “cryptic” lamellipodia (Farooqui and Fenteany, 2005) (Figure 5B).

Similar cryptic lamellipodia have also been observed in follower cells of the zebrafish lateral line migration model (Lecaudey et al., 2008; Dalle Nogare et al., 2014), thus suggesting that it might be a more general feature that people have previously realized. Importantly, however, we recently showed that the lamellipodia that mammary gland leader cells form are all hidden, cryptic ones; they are different from the typical lamellipodia sent into the matrix as observed in all of the other collective systems documented so far (Lu et al., 2020). Thus, “cryptic lamellipodia” may simply be a common feature of the cellular extensions formed by the cells that do not have a free edge. They are most likely the same as those typical lamellipodia and are based on the Arp2/3-mediated actin network.

Finally, while in most developmental settings, forces are generated on the basal side of the cells, it important to note that exceptions do exist. For example, during gastrulation of the red flour beetle, the apical domain of the blastoderm engages with the vitelline envelope, and the force hereby generated is essential for the morphological process to take place (Munster et al., 2019).

### Polarity Issues in Follower Cells

There are two issues, both concerning polarity in follower cells if we accept that they can generate a driving force for the migrating collective. First, how might front-rear polarity be set up in follower cells? Given our earlier discussions on how external cues specify leader cell fate and set up a polarity, it is safe to assume that a different polarity cue should be at work for follower cells. One way this could be accomplished is by using self-generated chemotactic gradients (Dona et al., 2013; Dalle Nogare et al., 2014; Muinonen-Martin et al., 2014). For example, leader cells in the zebrafish lateral line, which are stimulated by CXCL12/SDF-1, secret FGF which can themselves function as chemoattractants (Dona et al., 2013; Dalle Nogare et al., 2014). Thus, in theory, at least, FGF secreted by leader cells may function as a secondary gradient to set up follower cells’ front-rear polarity in the lateral line.

Another way to set up front-rear polarity in follower cells is by mechanical force-sensing. Although direct evidence is still lacking, recent studies based on 2D wound-healing assays show that cadherin-based cell-cell adhesion can mediate force propagation to set up polarity in follower cells (Cai et al., 2014; Plutoni et al., 2016). For example, Plutoni et al. showed that P-cadherin plays an essential role in mouse myoblasts-based 2D models to set up front-rear polarity during collective migration (Plutoni et al., 2016). Moreover, during endothelial sheet migration, front-rear polarity in leader cells and follower cells is determined by an FGF gradient and cadherin-associated proteins, respectively (Vitorino and Meyer, 2008). These findings thus correlate with the crucial roles of cadherins in follower cell polarity and with earlier studies showing that follower cells can actively migrate using similar mechanisms as leader cells, including dependence on Rac activation (Fenteany et al., 2000; Farooqui and Fenteany, 2005).

However, a second, and more important issue is how follower cells may have two polarities, namely apicobasal polarity and front-rear polarity, in the same cells. Up to this point, these two kinds of polarities are mutually exclusive in our discussion. Indeed, in all of the epithelial and epithelial-derived models that have currently been studied, a cell either has an apicobasal polarity, which makes it epithelial, or front-rear polarity, which makes it mesenchymal. This is understandable because the establishment and maintenance of both forms of polarity require the same set of regulators, which is the basis of why these two polarities are mutually exclusive in the systems we have examined thus far (Figure 5B).

Before any mechanistic insight can be provided, an important task toward understanding this interesting question is to validate in future studies that follower cells indeed have both apicobasal and front-rear polarities. This could be accomplished by a careful characterization of their organelles, cytoskeleton, and membrane proteins and lipids that are known to be differentially distributed in polarized cells. This should then be followed by determination of the subcellular localizations of components of the polarity machinery. The implication is that certain polarity components that mostly locate in only one domain under most conditions should now be found at, for example, both the apical and the front of follower cells. However, an alternative, and potentially more likely and more intriguing possibility is that a different set of polarity components may be employed in this unique situation.

Furthermore, an important question is how direction may be relayed from leader cells to follower cells, i.e., how is directional determination coupled between leader cells and follower cells. Interestingly, recent studies show that a wave of ERK signaling activation exists during collective migration. Its propagation from leader cells to follower cells appear to be essential for setting up directional information in the latter cells (Aoki et al., 2017; Hino et al., 2020). Moreover, cell-cell adhesion molecules, including both cadherins and catenins, may also play a role in this process, presumably via setting up a mechano-transduction signaling event (Bazellieres et al., 2015; Colak-Champollion et al., 2019; Khalil and de Rooij, 2019; Ozawa et al., 2020).

## Diverse Modes of Epithelial Migration

Based on the above discussion, it is clear that epithelial cells are much more migratory than historically recognized. Moreover, neither epithelial or mesenchymal state is static; rather, they are highly dynamic and can convert into one another given the right stimuli.

### Migration Is a Fundamental Cell Behavior

Modern technological advances have changed the way biology is studied. Long-term live imaging, for example, has given us an unprecedented view of the lives of epithelial cells that have eluded scientists until just a decade ago. The notion is now obsolete that epithelial cells, irrespective of whether they are from a mature epithelial tissue during postnatal homeostasis, but especially during embryonic development, are a static, immotile population of cells. Consistent with this notion, tight junctions, once thought to be stable structures that prevent epithelial cells from movements, are very dynamic and are under constant modulation and remodeling (Takeichi, 2014).

Epithelial motility is especially astounding during early embryonic development and organ formation. Indeed, long-range, large-scale migration of epithelial cells are evident during epiboly, in which ectodermal cells envelop the entire embryo, gastrulation, in which the previously separated ectoderm and endoderm are brought in contact with each other to generate mesoderm, convergent extension, in which mesodermal cells migrate toward each other to narrow one of their 3D axes while extending another, so on and so forth (Andrew and Ewald, 2009). In this sense, it is not an exaggeration to say that epithelial cells are born to run.

However, not all of the above modes of epithelial migration are directional, which is the focus of the current review. As such, they do not all involve a polarity change from apicobasal to front-rear polarity. Take convergent extension, for example, epithelial cells do not acquire front-rear polarity and their migration is instead regulated by components of planar cell polarity, the axis that organizes cells in the plane of the tissue (Andrew and Ewald, 2009). Despite a lack of direction as a collective during their migration, these epithelial morphological processes share many similarities with directional migration. For example, in almost all of these various processes, there is a tremendous amount of cell shape changes and movements or migration of individual cells within a given cell cluster (McShane et al., 2015; Diaz-de-la-Loza et al., 2018). Moreover, arrangements of both the apical and basal domains are also integrated into the overall morphological processes.

### EMT-Dependent and -Independent Modes of Migration

Although it was proposed to explain the puzzling observations where migrating cells expressing a mixture of both epithelial and mesenchymal signatures, based on the above discussions the term “partial EMT” suffers from the following issues. First, it was coined to explain how various epithelial collectives, as detailed in the current review, migrate even though they do not follow the traditional description of the EMT process. Despite this good intention, the scientists who coined the term were unaware at the time of an important characteristic of collective migration, i.e., most migrating epithelial collectives are a heterogeneous, rather than a homogeneous population as previously believed. Aside from leader cells, which have lost apicobasal polarity, follower cells, which consist of the majority of the cell population, are still epithelial, having apicobasal polarity, tight junctions, and all other epithelial-related features.

Interestingly, one of the recent breakthroughs in cancer biology is the recognition, thanks to the advancement in single-cell biology, that cancer cells are not homogenous as people previously believed, and cancer cell heterogeneity is essential for understanding every aspect of cancer biology, including drug resistance, and the development of novel therapeutic interventions (Vasan et al., 2019). Similarly, the use of “partial EMT” ignores, though unintentionally, the observation that migrating collectives are a mosaic and heterogeneous population and prevents a more thorough understanding of its mechanism.

A second issue with “partial EMT” is that it assumes that an EMT program can be best or accurately described, using a set of molecular signatures, at the level of gene expression. However, during collective migration, regardless whether EMT is involved, changes of gene expression levels, as emphasized by “partial EMT,” often do not matter nearly as much as how the essential regulators are changed in their subcellular locations, phosphorylation status, etc. Specifically, as discussed above, both epithelial and mesenchymal states show cell polarities, and both of which are regulated by the same set of regulators. Many of these regulators are either kinases or their functions change depending on their phosphorylation status (St Johnston and Ahringer, 2010). Thus, when epithelial and mesenchymal states convert, it is the intracellular locations and protein modification status of these polarity regulators and their targets that change, rather than their gene expression levels.

Finally, one of the basic differentiating factors of the epithelial or mesenchymal state is their migratory ability. As discussed above, this is mainly regulated at the level of cytoskeletal, and especially actin dynamics, which cannot be accurately described by changes in gene expression (Pollard and Borisy, 2003; Petrie et al., 2009; Vicente-Manzanares et al., 2009; Suraneni et al., 2012; Bravo-Cordero et al., 2013; Krause and Gautreau, 2014). Thus, while we recognize the historical roles that the EMT concept has played in our understanding of epithelial migration, we must also acknowledge that directional migration of the epithelium is dynamic and diverse, containing both EMT-dependent and -independent modes of migration. Together, we argue that the term “partial EMT” is a misnomer and its usage, especially regarding it being a mechanism by which collective epithelial migration occurs, should be discontinued.

## Conclusion

The discovery of EMT is a landmark event in the history of both developmental biology and cancer biology. It uncovered a recurring mode of epithelial migration that is central for the understanding of various key processes in these two disciplines. However, as a historical concept, it is not entirely congruent with recent progress in epithelial collective migration. In the current review, we discussed the root causes of the inconsistencies and controversies involving EMT and epithelial collective migration. We argue that the epithelial state is dynamic and there are diverse modes of epithelial migration, some are dependent on EMT whereas others are not.

We have come a long way in understanding epithelial motility, from our initial belief that an epithelium was a static, immotile tissue to one that is highly plastic, dynamic, and being able to engage in multiple modes of the migration process. While much of the advances can be attributed to the progress that has been made in our understanding of epithelial collective migration, many important questions remain unanswered. For example, although cell polarity in invertebrate systems is relatively well studied, it appears to be governed by a complex and previously unappreciated redundant mechanism in the vertebrate epithelium (Choi et al., 2019; Schmidt and Peifer, 2020). Moreover, consistent with being a part of the social collective, follower cells are increasingly thought to play an active role in collective migration (Venhuizen and Zegers, 2017). The exact mechanism, however, regarding how follower cells coordinate with leader cells has remained largely unclear.

The last decade has witnessed an incredible number of technological advances. CRISPR-mediated genome editing techniques have allowed us to study gene functions much more easily than before, while the development of a large array of biosensors, including those for GTPases, mechanical forces, and cytoskeletal dynamics, etc. have made it possible to capture key events during epithelial migration. We await with great anticipation the next phase of tremendous progress in this exciting area.

## Author Contributions

Both authors conceived topics and wrote the manuscripts together.

## Conflict of Interest

The authors declare that the research was conducted in the absence of any commercial or financial relationships that could be construed as a potential conflict of interest.

## Publisher’s Note

All claims expressed in this article are solely those of the authors and do not necessarily represent those of their affiliated organizations, or those of the publisher, the editors and the reviewers. Any product that may be evaluated in this article, or claim that may be made by its manufacturer, is not guaranteed or endorsed by the publisher.
